# Levosimendan: A Cardiovascular Drug to Prevent Liver Ischemia-Reperfusion Injury?

**DOI:** 10.1371/journal.pone.0073758

**Published:** 2013-09-11

**Authors:** Peter Onody, Rita Stangl, Andras Fulop, Oliver Rosero, David Garbaisz, Zsolt Turoczi, Gabor Lotz, Zoltan Rakonczay, Zsolt Balla, Viktor Hegedus, Laszlo Harsanyi, Attila Szijarto

**Affiliations:** 1 1^st^ Department of Surgery, Semmelweis University, Budapest, Hungary; 2 2^nd^ Department of Pathology, Semmelweis University, Budapest, Hungary; 3 First Department of Medicine, University of Szeged, Szeged, Hungary; New York Medical College, United States of America

## Abstract

**Introduction:**

Temporary occlusion of the hepatoduodenal ligament leads to an ischemic-reperfusion (IR) injury in the liver. Levosimendan is a new positive inotropic drug, which induces preconditioning-like adaptive mechanisms due to opening of mitochondrial K_ATP_ channels. The aim of this study was to examine possible protective effects of levosimendan in a rat model of hepatic IR injury.

**Material and Methods:**

Levosimendan was administered to male Wistar rats 1 hour (early pretreatment) or 24 hours (late pretreatment) before induction of 60-minute segmental liver ischemia. Microcirculation of the liver was monitored by laser Doppler flowmeter. After 24 hours of reperfusion, liver and blood samples were taken for histology, immuno- and enzyme-histochemistry (TUNEL; PARP; NADH-TR) as well as for laboratory tests. Furthermore, liver antioxidant status was assessed and HSP72 expression was measured.

**Results:**

In both groups pretreated with levosimendan, significantly better hepatic microcirculation was observed compared to respective IR control groups. Similarly, histological damage was also reduced after levosimendan administration. This observation was supported by significantly lower activities of serum ALT (p_early_ = 0.02; p_late_ = 0.005), AST (p_early_ = 0.02; p_late_ = 0.004) and less DNA damage by TUNEL test (p_early_ = 0.05; p_late_ = 0.034) and PAR positivity (p_early_ = 0.02; p_late_ = 0.04). Levosimendan pretreatment resulted in significant improvement of liver redox homeostasis. Further, significantly better mitochondrial function was detected in animals receiving late pretreatment. Finally, HSP72 expression was increased by IR injury, but it was not affected by levosimendan pretreatment.

**Conclusion:**

Levosimendan pretreatment can be hepatoprotective and it could be useful before extensive liver resection.

## Introduction

The liver is susceptible to numerous conditions associated with hypoxia or hypoperfusion. During extensive liver resections, temporary occlusion of the hepatoduodenal ligament – widely known as Pringle’s maneuver - is often used to control bleeding [1]. However, this maneuver can lead to ischemic-reperfusion (IR) injury of the liver. Recently, a total exclusion of the hepatic inflow is rarely necessary due to more advanced bleeding control and operative techniques. However, inflow exclusion of the portal vessels may be unavoidable if unexpected hemorrhage occurs during traumatic liver injury or transplantation.

A large number of studies investigated various methods how to attenuate IR injury in the liver. Of those, the most frequently investigated is ischemic preconditioning (IP), which seems to be the most effective, too [2], [3]. The hepatoprotective effect of IP can be detectable in two distinct patterns (two windows of protection) in terms of time course. The first, which is known as “early” preconditioning lasts for 1–2 hours. The second is usually referred as “late” preconditioning, and it begins 24 hours subsequent to the conditioning stimulus and lasts up to 48–72 hours thereafter [4], [5]. A better understanding of the underlying signaling pathways made it possible to apply various pharmacological agents to induce hepatoprotection against IR experimentally [6].

Mitochondria play key roles in cellular IR injury, due to their crucial functions in energy production and programmed cell death. A dominant factor in mitochondrial damage and subsequent dysfunction is the opening of the mitochondrial permeability transition pores (MPTP) located in the inner membrane of the organelle [7], [8]. The mitochondrial adenosine triphosphate-dependent potassium channels (mito-K_ATP_) have critical effect in regulating mitochondrial volume as well as function [9]. Inhibition of mito-K_ATP_ channels leads to suspension of the protective effect of IP, whereas channel-opening chemical compounds can provide protection against IR injury similar to IP. It is assumed that mito-K_ATP_ may be able to prevent long-term opening of MPTP, thus preserving the integrity of the mitochondria and ensuring a better cellular energy status. Based on the above, chemical induction of mito-K_ATP_ opening can be a potential mechanism for pharmacological preconditioning [10], [11].

Levosimendan is an inodilator, cardioprotective drug used in the management of acute heart failure. This agent exerts a positive inotropic and an anti-stunning effect by increasing calcium sensitivity of the myocardial contractile elements, as well as a vasodilatator effect by opening sarcolemmal K_ATP_ channels in vascular smooth muscle cells. Recent studies demonstrated that levosimendan is able to open the mito-K_ATP_ channels, too [12]. These results prompted *in vitro* and *in vivo* studies on the anti-ischemic effect of the drug, suggesting that levosimendan has a direct cellular protective effect against IR injury [13]. Further, levosimendan does not reduce splanchnic blood flow in contrast to other positive inotropic agents, and it has a positive effect on small bowel and liver perfusion, too [14] [15]. In addition, it was demonstrated that levosimendan can protect against acute renal failure in sepsis [16].

Therefore, we aimed to study the protective effect of levosimendan against liver IR injury in an experimental rat model.

## Materials and Methods

### Animals

Male Wistar rats, weighing 250–280 g were used in the experiments (Charles River Hungary Ltd.). The experimental design was regulated by Act XXVIII of 1998 and Government Decree 243/1998 (XII. 31), and approved by committee on Animal Experimentation of Semmelweis University (license number: 22.1/743/001/2007). The rats were kept on standard chow and water *ad libitum* under specific, pathogen-free conditions at 22–24°C. For 12 hours prior to surgery water was provided only. Each experiment was started at the same time of the day to avoid any possible effects of the circadian rhythm.

### Pretreatment Protocol

Levosimendan pretreatment was applied 1 or 24 hours before the induction of liver IR injury to mimic the two distinct patterns in time for therapeutic effect of surgical ischemic preconditioning. Levosimendan (Simdax®, OrionPharma Ltd, Hungary) was administered as a total dose of 54 µg/kg in 5 cycles (each cycle for 5 min) dissolved in 5% glucose solution via a polyethylene catheter placed into the left jugular vein (PolyE Polyethylene Tubing, Harvard Apparatus, United States). A 10 minutes pause was held between infusion cycles to create a pattern similar to IP. Control and sham-operated animals received the vehicle in the same pattern.

### Operative Procedure

Animals were anaesthetized with intraperitoneal injections of ketamine (75 mg/kg) and xylazine (7.5 mg/kg). Deep anesthesia was maintained by intravenous administration of 25 mg/kg/h ketamine and 2.5 mg/kg/h xylazine via a 22-gauge polyethylene catheter placed into the right jugular vein. Another polyethylene catheter was inserted into the femoral artery to monitor mean arterial blood pressure (MAP) and heart rate (HR) (Kent Scientific Corporation, Torrington, CT, USA). The animals were allowed to breathe spontaneously during surgery. Intraoperative normothermia (36.5–37.5°C) was maintained by a heating pad connected to a rectal thermometer.

**Figure 1:**
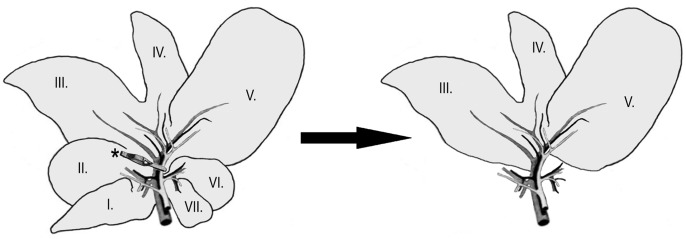
Liver lobes III, IV, V were subjected to 60(asterix). Immediately before reperfusion, the shunting lobes (I, II, VI, VII) were removed, thus reperfusion affected only the post-ischemic tissue.

A standardized surgical model was used for assessment of liver IR damage as described previously [17], [18], [19]. (Figure 1) Briefly, after median laparotomy and mobilization of the liver, lobes III, IV, V were subjected to 60 min ischemia by clamping of the biliovascular trunk using an atraumatic microvascular clip. Immediately before reperfusion, the shunting lobes (I, II, VI, VII) were removed, thus reperfusion affected only the post-ischemic tissue (65–70% of the total hepatic mass). The microcirculation of lobe V was monitored using laser Doppler flowmeter (LDF) throughout the ischemic period and the first hour of reperfusion. During IR periods, the abdomen was covered with a plastic wrap to minimize ﬂuid loss via evaporation. At the end of the first hour of reperfusion the abdomen was closed and the animals were returned to their cages. After 24 hours reperfusion, animals were anesthetized with intraperitoneal injection of ketamine (75 mg/kg) and xylazine (7.5 mg/kg) and were sacrificed by exsanguinations via right ventricular puncture, then blood and histological samples were taken.

### Experimental Groups

A total of 55 animals were randomly separated into two main groups: **(E)** “**early**” (pretreatment 1 h before surgery) and **(L)** “**late**” (pretreatment 24 h before surgery).

**(S)** **Sham-operated group (n = 5):** rats were subjected to glucose pretreatment (as detailed in pretreatment protocol section) and surgical procedures (as described above), except for induction of liver ischemia, but including liver resection (lobes I, II, VI, VII).

**(C_E_; C_L_) Control groups (n = 5–5):** rats – similarly to the sham-operated group – were subjected to the surgical procedures as well as to “early” or “late” levosimendan pretreatment.

**(IR_E_; IR_L_) Ischemia-reperfusion groups (n = 10–10):** animals underwent the entire surgical procedure, including the 60 minutes partial liver ischemia and liver resection followed by 24 h of reperfusion.

***(L_E_; L_L_) Levosimendan*** **pretreated groups (n = 10–10):** rats received levosimendan 1 h or 24 h prior to liver IR and were operated similarly to the IR group.

### Assessment of Hepatic Microcirculation

Liver microcirculation was evaluated by laser Doppler flowmeter (Moor Instruments Ltd, London, UK). The LDF probe was placed at the same position on lobe V in every experiment. For characterization of the individual ﬂow graphs, a mathematical correction was performed as described previously by us. To compare the flow graphs, the integral of the reperfusion segment of the graphs (RA: reperfusion area) and the maximal plateau of the last 10 minutes of the reperfusion (PM: plateau maximum) were used [20].

### Histopathological Analysis

Methods of histopathological analysis were based on our previous publications [18], [19]. Samples from excised lobes III, IV, V were fixed in 4% neutral-buffered formalin for 24 hours, dehydrated and embedded in paraffin. Sections of 3–5 µm thickness were stained with hematoxylin and eosin (H&E). During histological evaluation, the following changes were evaluated by an experienced pathologist: (1) cellular swelling, (2) lipoid degeneration, (3) sinusoidal congestion, (4) tissue hemorrhage, (5) leukocyte infiltration, (6) necrosis and (7) signs of apoptosis. These pathological changes were semiquantitatively scored as follows: 0: no alteration, +: <10% of affected cells, ++: <50% of affected cells, +++: >50% of affected cells. Hence, the overall maximum score was 21. The evaluating pathologist was blinded to the experiment.

### Immunohistochemical Analysis

Terminal deoxynucleotidyl transferase-mediated dUTP nick endlabeling (TUNEL) assay was used to further assess extent of damaged areas. Commercially available kits were used (Chemicon International Inc, Temecula, CA, USA), and histological slides were counterstained with hematoxylin (Vector Laboratories, Burlingame, CA, USA). The size of the demarcated TUNEL positive areas was evaluated and expressed as a percentage of the whole section.

Poly(ADP-ribose) polymerase (PARP) activation was measured by immunohistochemical detection of the enzyme’s product, poly(ADP-ribose) (PAR) with the use of mouse monoclonal anti-poly(ADP-ribose) antibody (1∶1000, Calbiochem), as described previously [21]. Immunoreactivity was evaluated in the demarcated areas as well as in the surrounding areas; the ratio of PAR positivity is shown as a percentage value.

### Measurement of Serum ALT and AST

Blood samples were centrifuged (1050 g for 2×10 min, at room temperature) and the supernatant was collected. Serum samples were frozen in liquid nitrogen and stored at −80°C. Alanine aminotransferase (ALT) and aspartate aminotransferase (AST) were quantified by standard spectrophotometry using automated clinical chemistry analyzer (Hitachi 747, Hitachi Ltd, Tokyo, Japan).

### Measurement of Antioxidant Status

Total scavenger capacity in the plasma (blood samples were centrifuged at 1050 g for 2×10 min at 4°C) and liver homogenates were measured in H_2_O_2_/OH^•^ luminol microperoxidase system using Lumat LB 9051 luminometer (Lumat; Berthold, Windbad, Germany) [22]. The chemiluminescence light intensity - given in relative light units (RLU) - was proportional to the concentration of free radicals. The results were expressed as a percentage compared to the background (RLU %). Protein content was measured using Lowry’s method [23].

Free SH-groups were detected using the Sedlak method based on Ellmann reaction [24]. The results show the protein-related reducing power in mmol/L. The H-donating ability, reflecting the non-protein-bound antioxidant state of the samples, was measured in the presence of a 1,1-diphenyl-2-picryl-hydrasyl radical at 517 nm using Blois’ method as modified by Blázovics et al [25], [26]. The results were expressed in percentage of inhibition. The samples’ reducing power (RP) was assessed using Oyaizu’s method [27]. The changes in absorbance caused by transformation of Fe^3+^ into Fe^2+^ were detected at 700 nm and compared with the changes of ascorbic acid (AA). The spectrophotometric measurements were carried out with Jasco V-550.

Luminol, microperoxidase, hydrogen peroxide were purchased from Sigma (St. Louis, MO, USA), the other chemical reagents were obtained from Reanal Chemical Co. (Budapest, Hungary).

### Liver Tissue Viability

Parts of lobe V were frozen in liquid nitrogen and stored at −80°C. Five mm thick cross-sections were made. Slides were incubated for 30 min at 37°C in nitroblue tetrazolium (NBT, 18 mg/l) and NADH (150 mg/l) reagents (Sigma-Aldrich Inc, St. Louis, MO, USA) diluted in 0.05 M TRIS buffer (pH 7.6). Unused tetrazolium reagent was removed by ascending (30%, 60% and 90%), followed by descending concentrations of acetone [28]. The amount of colored reaction product was directly proportional to the absolute number of the functional mitochondrial NADH-dehydrogenase enzyme complex, it could therefore be used to determine mitochondrial integrity and cell viability.

Viability was assessed by quantitative evaluation of the reaction. Ten random fields were microphotographed. The amount of generated reaction product was determined using Leica Qwin Pro image analysis software (Leica Microsystems Imaging Solutions Ltd, Cambridge, UK). The obtained amount was then compared to the total area. All viewing fields were evaluated separately. Regarding whole sample, the ratio was calculated as a ten-field-average and expressed as a percentage of NBT positivity of simple sham-operated animals.

### Heat Shock Protein (HSP) 72 Expression

HSP72 expression of the liver was measured from tissue homogenate using Western blot analysis [29]. The bands were visualized by chemiluminescence technique. Detection and quantitative analysis of results were achieved using ImageJ software (NIH, Bethesda, MD, USA).

### Statistical Analysis

Values were expressed as means ± SD. Statistical significance was determined by one-way analysis of variances (ANOVA) followed by Scheffer’s post-hoc test. A p<0.05 confidence interval was considered as statistically significant.

## Results

### Hemodynamic Parameters

Immediately (within 1 min) after the levosimendan pretreatment, there was a significant decrease in the mean arterial blood pressure (p = 0.044) and an increase in the heart rate (p = 0.049) as compared to groups receiving glucose only. The blood pressure measured directly before the ischemic period was similar to the initial value in the “late” groups.

Hemodynamic parameters did not change significantly in any of the experimental groups throughout the 60 minutes of ischemia. After induction of reperfusion, tachycardia and a considerable reduction of MAP (p_IRE_ = 0.047; p_IRL_ = 0.033) were observed in the IR animals (IR_E_; IR_L_). 5–10 minutes after the onset of reperfusion blood pressure normalized slowly, but it did not reach the pre-ischemic values completely. In contrast, reperfusion did not cause further significant drop in the blood pressure in the levosimendan pretreated groups, furthermore, MAP reached the baseline level at the end of the first hour of reperfusion.

### Microcirculation Measured by LDF

**Figure 2:**
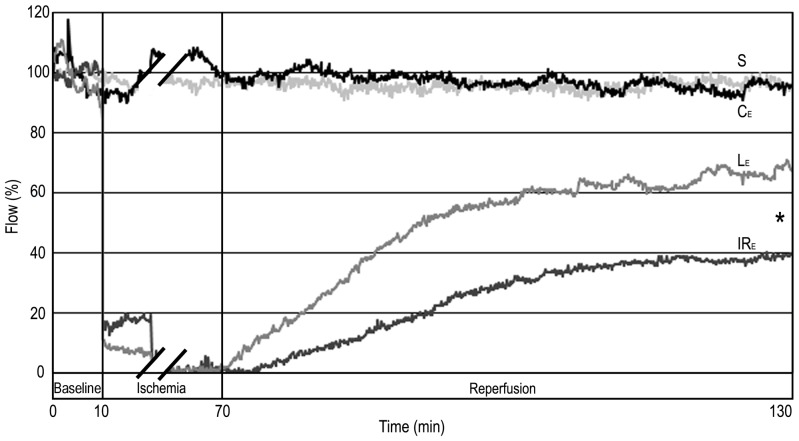
Hepatic microcirculation after “early” levosimendan pretreatment. The blood flow of sham-operated (S) and “early” control group (C_E_) did not change significantly. There was a decline of the ﬂux in groups subjected to IR (IR_E_; L_E_). Levosimendan pretreatment (L_E_) significantly improved liver microcirculation compared to the IR_E_ group during reperfusion. Values are expressed as means. * p<0.05 versus IR_E_ group. n = 5 in sham-operated (S) and control groups (C_E_); n = 10 in IR (IR_E_) and levosimendan pretreated groups (L_E_).

**Figure 3:**
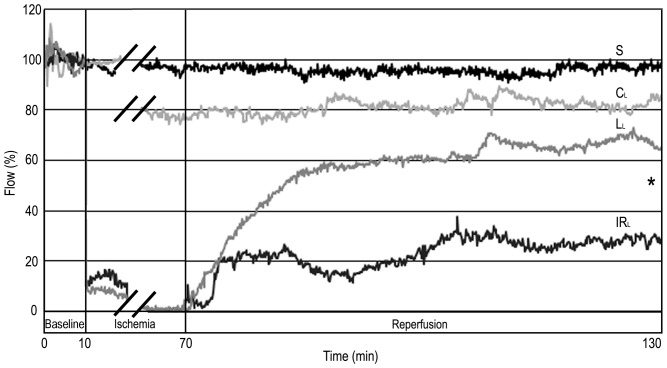
Hepatic microcirculation after “late” levosimendan pretreatment. In the “late” control group (C_L_) a reduction of blood flow was observed in comparison to sham-operated animals (S), but the difference was not significant. However, there was a significant decline of the ﬂux in groups subjected to IR (IR_L_; L_L_). Levosimendan pretreatment (L_L_) caused significant improvement in the microcirculation of the liver compared with the IR_L_ group. Values are expressed as means. * p<0.05 versus IR_L_ group. n = 5 in sham-operated (S) and control group (C_L_); n = 10 in IR (IR_L_) and levosimendan pretreated group (L_L_).

Both “late” and “early” levosimendan pretreatments caused significant improvement (RA: p_early_ = 0.0012; p_late_ = 0.0010; PM: p_early_ = 0.0019; p_late_ = 0.0007) in the microcirculation of the liver compared with the respective IR groups (Figures 2–3 & Table 1).

**Table 1:** Microcirculatory data of the liver.

Microcirculatory parameters	Experimental groups						
		“early” pretreatment			“late” pretreatment		
	S	C_E_	IR_E_	L_E_	C_L_	IR_L_	L_L_
**Reperfusion area (RA) %**	95.7±5	97.3±22	23.9±12	47.2*±6	81.3±14	22.8±10	55.6*±18
**Plateau maximum (PM) %**	95.6±5	100±24	37.9±16	66.8*±9	81.8±12	28.4±9	67.1*±22

S: sham-operated; C_E_: “early” control; IR_E_: “early” ischemia-reperfusion; L_E_: “early” levosimendan pretreated; C_L_: “late” control; IR_L_: “late” ischemia-reperfusion; L_L_: “late” levosimendan pretreated.

* p<0.05 versus the respective IR group.

### Histopathological Analysis

There was no pathological change detectable in the “early” control group (C_E_), except occasional mild sinusoidal dilatation (total score: 2.1). In the IR group (IR_E_), however, necrotic areas, substantial periportal lymphocyte infiltration and tissue hemorrhage were found (total score: 11.6). In the levosimendan pretreated group (L_E_) significantly less focal necrosis was seen (total score: 7.8; p = 0.043). Tissue hemorrhage was not typical and leukocyte infiltration was less extensive.

**Figure 4:**
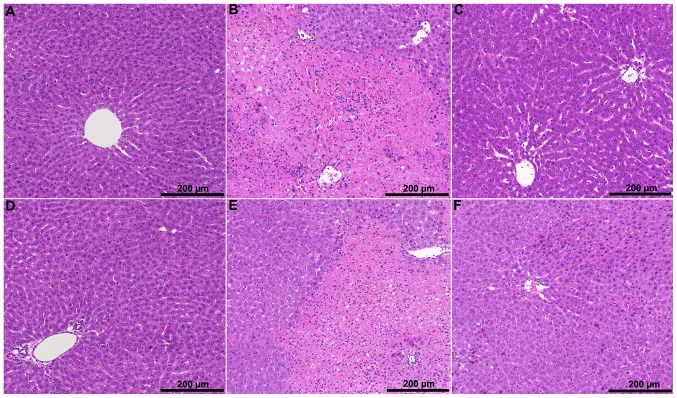
Representative H&E-stained liver sections. In the control groups (A: “early” control; D: “late” control) mild tissue injury and sinusoidal dilatation were observed. In the IR groups (B: “early” IR; E: “late” IR) confluent necrotic areas were detected accompanied by significant leukocyte infiltration and tissue hemorrhage. The levosimendan pretreated groups (C: “early” levosimendan pretreatment; F: “late” levosimendan pretreatment) were characterized by focal necrosis associated with milder tissue hemorrhage and less severe leukocyte infiltration.

In the “late” control group (C_L_), increased sinusoidal dilatation and occasional perivascular edema were observed when compared with the sham-operated and “early” control groups (total score: 4.8). In the IR group (IR_L_) extensive, predominantly panlobular necrosis was detected, which was associated with significant leukocyte infiltration and tissue hemorrhage (total score: 12.3). The levosimendan pretreated group (L_L_) was characterized by focal necrosis, milder tissue hemorrhage and less severe inflammatory cell infiltration (total score: 7.9; p = 0.041) (Figure 4).

### Immunohistochemical Analysis

After liver injury induced by IR, almost exclusively demarcated areas of positive cells were observable with TUNEL immunohistochemical staining. After levosimendan pretreatment, however, a significant reduction of the demarcated areas was seen both in the “early” and the “late” groups, when compared to the corresponding IR groups (p_early_ = 0.05; p_late_ = 0.034).

Furthermore, PAR-positive area was significantly reduced, too, after “late” levosimendan pretreatment compared to the “late” IR group (p_late_ = 0.04) (Table 2).

**Table 2:** Immunohistochemical analysis.

Measured parameters	Experimental groups						
		“early” pretreatment			“late” pretreatment		
	S	C_E_	IR_E_	L_E_	C_L_	IR_L_	L_L_
**TUNEL positivity**	0	0	5.7±0.9	1.2±0.6	0	15.7±1.3	1.2±0.7
**in the demarcated area (%)**							
**PAR positivity**	–	–	6.3±0.8	1.5±0.3	–	17.3±1.9	1.6±0.5
**in the demarcated area (%)**							

S: sham-operated; C_E_: “early” control; IR_E_: “early” ischemia-reperfusion; L_E_: “early” levosimendan pretreated; C_L_: “late” control; IR_L_: “late” ischemia-reperfusion; L_L_: “late” levosimendan pretreated.

### Measurements of Serum ALT and AST

Serum ALT activity in the IR groups was significantly higher than the sham-operated groups. However, serum ALT activity was significantly lower in the levosimendan pretreated groups than the IR groups (p_early_ = 0.02; p_late_ = 0.005).

**Figure 5:**
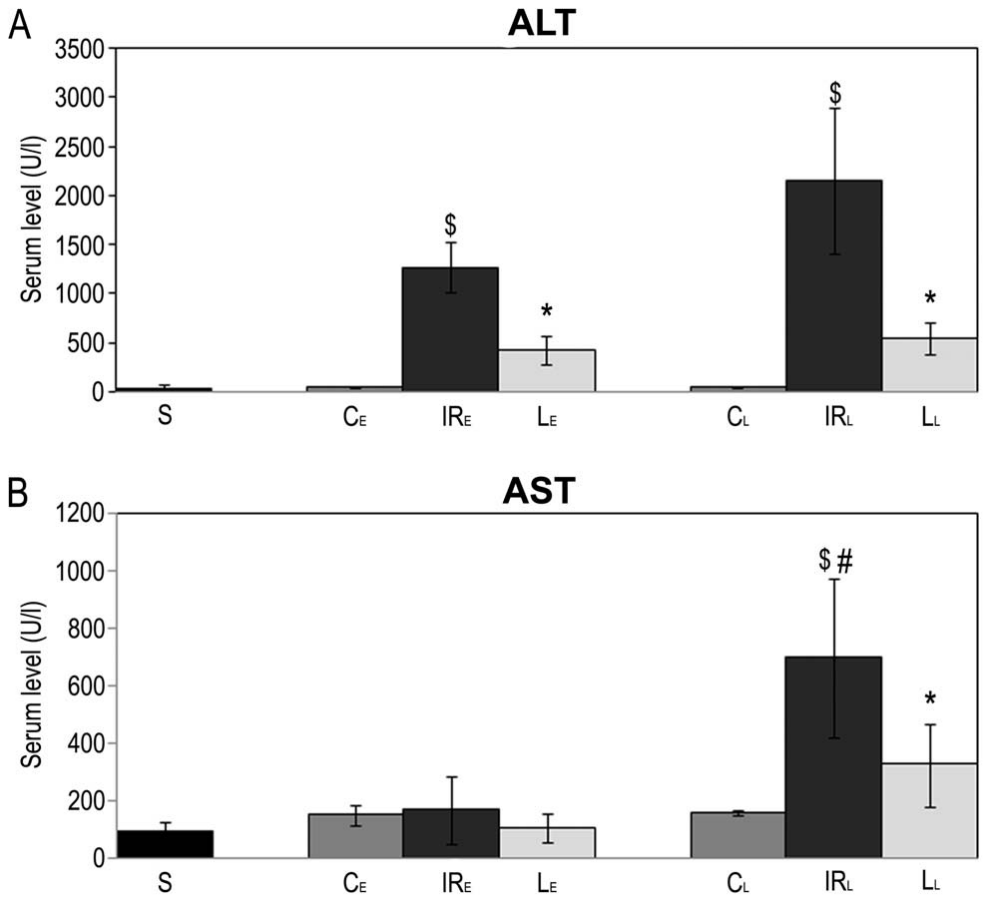
Serum level of ALT and AST. Ischemic-reperfusion injury of the liver led to an increase in serum activities of alanine aminotransferase (ALT) and aspartate aminotransferase (AST).A: Serum levels of ALT significantly decreased in the levosimendan pretreated groups (L_E_, L_L_) compared to the corresponding IR groups (IR_L_, IR_E_) B: Raised AST activity in the “late” IR group (IR_L_) were significantly higher than in the “early” IR group (IR_E_). “Late” levosimendan pretreatment significantly reduced the serum activity of AST. Data are shown as means+SEM, * p<0.05 versus “late” IR group; ¤ <0.05 versus “early” IR group; $ p<0.05 versus “late” control group; & p<0.05 versus “early” control group; # p<0.05 versus “early” IR group. n = 5 in sham-operated (S) and control groups (C_E,_ C_L_); n = 10 in IR (IR_L_, IR_E_) and levosimendan pretreated groups (L_E_, L_L_).

Serum AST activity in the “late” IR group (IR_L_) was higher than the “early” IR group (IR_E_). However, “late” levosimendan pretreatment substantially reduced serum AST activity (p_late_ = 0.04) (Figure 5).

### Measurement of Antioxidant Status

Free radical concentrations were significantly increased in the IR groups compared with sham operated and control animals. “Early” levosimendan pretreatment resulted in significantly lower RLU% values in the serum after 24 hours of reperfusion when compared with the IR groups (p_early_ = 0.03).

Levosimendan pretreatment led to a significant improvement in the reducing power of the serum compared with the IR groups (p_early_ = 0.01; p_late_ = 0.03). In case of the liver, the improvement was significant only with the use of the “early” pretreatment protocol (p_early_ = 0.01).

Concentration of free SH-groups was significantly decreased in the liver in the levosimendan pretreated groups (p_early_ = 0.02; p_late_ = 0.03), whereas in serum samples only an improving tendency could be seen.

The non-protein-bound antioxidant capacity indicator H-donating ability showed significant improvement in the serum after “early” levosimendan pretreatment as compared with the IR groups (p_early_ = 0.04) (Table 3).

**Table 3:** Measurement of antioxidant status.

Measurement	Sample	Experimental groups								
		S	“early” pretreatment				“late” pretreatment			
			C_E_	IR_E_	L_E_	P*	C_L_	IR_L_	L_L_	P**
**Total scavenger** **capacity** (RLU%)	**serum**	4.38±1.68	4.4±2.15	12.07±2.77	8.51±2.62	**0.03**	3.77±3.03	9.69±2.35	6.71±3.74	0.06
	**liver**	21.25±1.95	28.35±18.55	82.15±13.24	66.73±4.63	0.06	24.39±6.74	75.12±17.15	56.34±7.34	0.07
**Reducing power**	**serum** (µmolAA/ml)	–	0.38±0.23	0.54±0.13	0.79±0.20	**0.01**	1.13±0.53	0.66±0.34	1.08±0.13	**0.03**
	**liver** (µmolAA/g prot)	393.6±45.3	266.4±34.0	148.6±41.1	220.1±22.5	**0.01**	317.8±16.6	207.4±27.5	248.7±36.4	0.06
**Free SH-groups**(mmol/l)	**serum**	–	0.043±0.018	0.023±0.007	0.030±0.007	0.06	0.045±0.006	0.023±0.009	0.034±0.019	0.07
	**liver**	0.077±0.02	0.063±0.015	0.041±0.011	0.061±0.018	**0.02**	0.055±0.008	0.043±0.016	0.051±0.012	**0.03**
**H-donating ability**	**serum**	–	64.01±8.95	40.66±9.38	51.28±7.03	**0.04**	58.01±9.20	38.75±8.02	47.83±11.05	0.08
	**liver**	44.35±4.95	40.94±6.33	14.77±2.61	18.68±3.52	0.06	45.37±6.96	18.74±2.49	23.13±4.48	0.07

S: sham-operated; C_E_: “early” control; IR_E_: “early” ischemia-reperfusion; L_E_: “early” levosimendan pretreated; C_L_: “late” control; IR_L_: “late” ischemia-reperfusion; L_L_: “late” levosimendan pretreated; AA: ascorbic acid;

* IR_E_ versus L_E_;

** IR_L_ versus L_L_.

### Liver Tissue Viability

**Figure 6:**
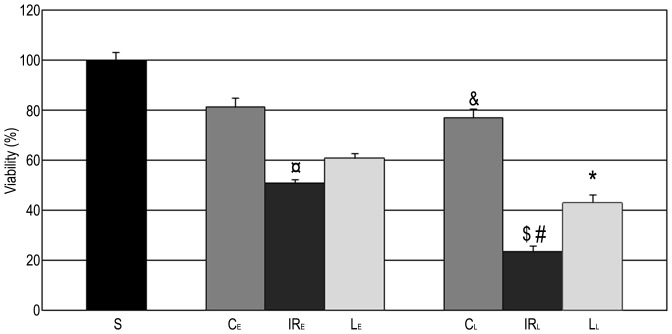
Liver tissue viability. Nitroblue tetrazolium (NBT) intensity of the “late” and “early” control animals was lower compared to the sham-operated group (S), but the difference was significant in the “late” category (C_L_) only. After IR injury, number of functioning mitochondria further decreased. NBT positivity was significantly lower in the „late” IR group (IR_L_) than in the „early” IR group (IR_E_). However, “late” levosimendan pretreatment was able to enhance significantly the number of viable mitochondria. The data are presented as means+SEM. ¤ <0.05 versus “early” control group; &p<0.05 versus sham-operated group; $ p<0.05 versus “early” IR group; #p<0.05 versus “late” control group; * p<0.05 versus “late” IR group. n = 5 in sham-operated (S) and control groups (C_E_, C_L_); n = 10 in IR (IR_L_, IR_E_) and levosimendan pretreated groups (L_E_, L_L_).

In the “late” control group, liver tissue was significantly less viable then sham-operated animals, and a similar tendency was observed in the “early” control group (S: 100%; C_E_: 82%; C_L_: 77%). Further, liver tissue turned out to be significantly less viable in the “late” IR control group compared to the “early” counterpart (IR_L_: 23%; IR_E_: 51%; p = 0.0001). Importantly, levosimendan pretreatment administered 24 hours before surgery significantly increased the proportion of NBT-positive areas (L_L_: 42%; p_late_ = 0.003), while “early” administration of levosimendan a similar effect, too (Figure 6).

### HSP72 Expression

**Figure 7:**
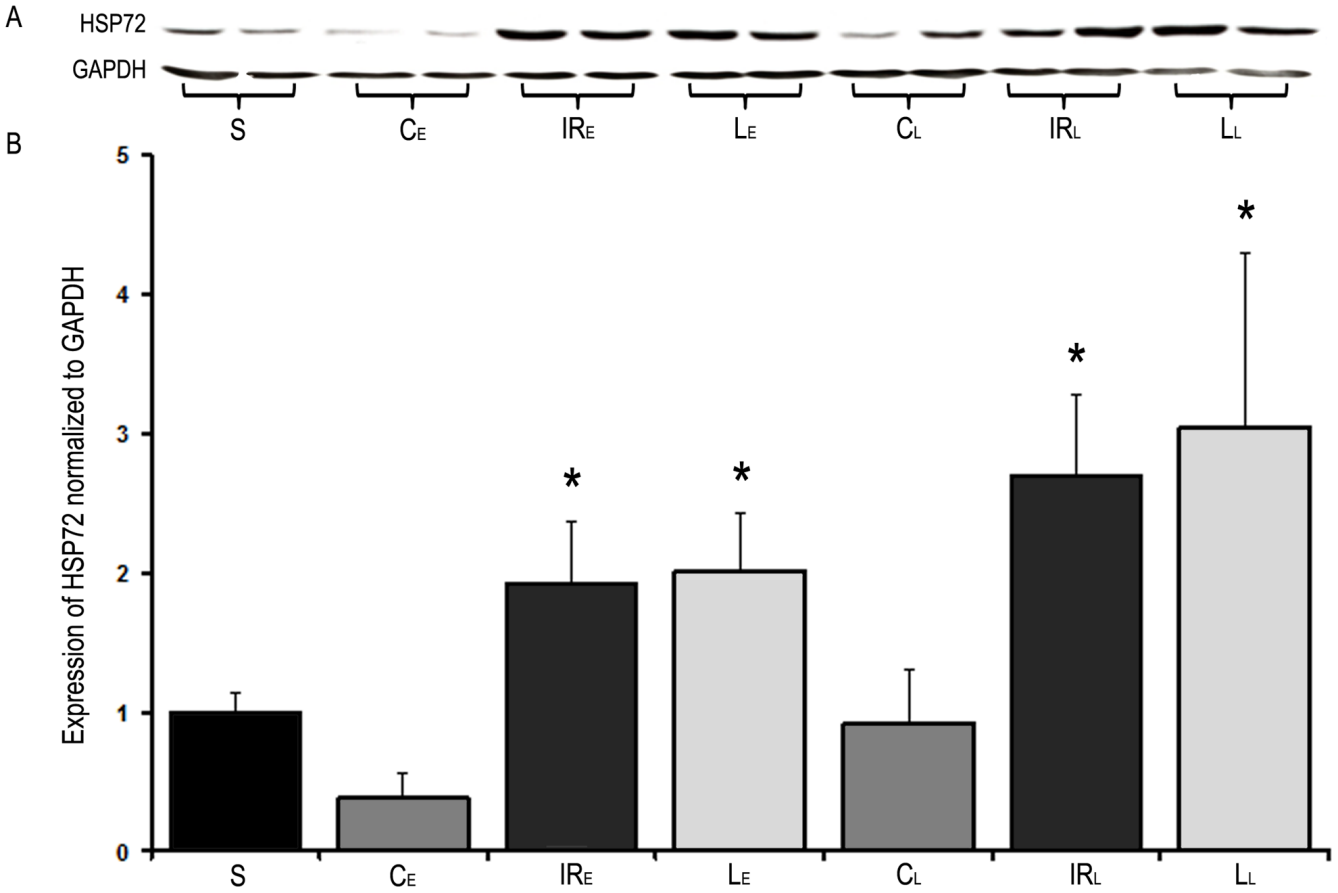
Liver HSP72 expression. **A:** Representative Western blotting for HSP72 in sham-operated group (S), control groups (C_E_, C_L_), IR groups (IR_E_, IR_L_) and levosimendan pretreated groups (L_E_, L_L_). **B:** Quantitative results of Western blotting. A significant increase in liver HSP72 expression was observed in the IR groups as well as in the levosimendan pretreated groups compared to the sham-operated group. Levosimendan pretreatment did not result in changes of HSP72 expression pattern. Data are presented as means+SEM, * p<0.05 versus sham-operated group. n = 5 in sham-operated (S) and control groups (C_E_, C_L_); n = 10 in IR (IR_L_, IR_E_) and levosimendan pretreated groups (L_E_, L_L_).

In both IR groups a substantial increase in liver HSP72 expression was observed compared with the sham-operated group. Neither the “early”, nor the “late” levosimendan pretreatment resulted in changes of the IR-induced HSP72 expression pattern (Figure 7).

## Discussion

Liver IR injury develops primarily during transplantation, traumatic injury or extensive liver resection due to a temporary occlusion of the hepatoduodenal ligament. Several methods have been tried to attenuate or prevent IR injury, but no significant success has been achieved until Murry published a new pioneering technique called ischemic preconditioning [30]. A better understanding of the underlying signaling mechanisms of IR-related pathological changes opened new perspectives in research focusing on treatment strategies for IR liver injury.

Levosimendan is a unique positive inotropic molecule in terms it does not reduce splanchnic circulation as well as it has anti-ischemic properties by opening mito-K_ATP_ channels [15] [31]. Hence, we examined the effect of levosimendan in a rat liver IR model.

Microcirculation is a crucial factor in IR liver injury. Changes in the microcirculatory blood flow usually precede the development of parenchymal abnormalities [32]. Microcirculatory changes may prolong ischemic time and enlarge irreversibly damaged areas. In addition, it can trigger progressive inflammatory response [33]. We demonstrated previously that improvement in microcirculation reduced hepatic injury [20]. Therefore, the quality of tissue microcirculation may indicate the severity of organ damage and the efficacy of any intervention. Literature data suggested that levosimendan improves microcirculatory blood flow of the splanchnic area in septic rats [34]. In consistent with the above, our results showed that levosimendan pretreatment applied 1 or 24 hours prior to surgery resulted in highly significant improvement in liver microcirculation compared with the corresponding IR groups.

In terms of H&E-stained histological slides, important differences were detected between the experimental groups. In the IR groups, large and often confluent areas of necrosis was observed, which was accompanied by significant hemorrhage and leukocyte infiltration. Meanwhile the levosimendan pretreated animals showed dramatically less cell death, which was mostly focal. Consistently, tissue bleeding was not typical and leukocyte infiltration was less extensive, too. The moderate tissue damage of pretreated animals was supported by a significant decrease in serum ALT and AST activities.

During IR liver injury, cell death is characteristic for hepatocytes and sinusoidal endothelial cells predominantly. Theoretically, cell death can happen as oncotic necrosis and apoptosis. Terminal deoxynucleotidyl transferase-mediated deoxyuridine triphosphate nick-end labeling (TUNEL) - commonly used to determine single- or double-strand DNA breaks – typically characterizes apoptotic cell death. However, DNA degradation occurs during necrosis, too, especially during IR damage due to nucleases released from inflammatory cells [35]. Therefore, this assay is not reliable to demonstrate apoptosis specifically, it is rather suitable to determine the extent of DNA damage as a cytotoxic consequence of IR [36]. Consistently, diffuse TUNEL positive areas were detected in the IR groups. These TUNEL positive areas corresponded to the extensively damaged parts seen in H&E-stained slides, where apoptosis and necrosis are likely to occur. After “early” and “late” levosimendan pretreatment, a significant decrease was observed in the size of TUNEL positive areas. These results are supported by literature data showing anti-apoptotic properties of levosimendan in other organs like the heart and kidney [37], [38]. Low level of DNA cleavage is supported by PAR-positivity of the demarcated region as well. PARP activity is a marker of DNA damage and repair, which is characteristic to excessive DNA damage [39]. PAR-positivity suggested a significantly lower DNA- and cell injury in the “late” levosimendan pretreated groups.

The ischemic insult leads to sublethal cell injury, which is exacerbated by acute generation of reactive oxygen species following reoxygenation. Free radicals cause direct tissue injury and initiate a number of noxious cellular responses leading to the formation of proinflammatory mediators and infiltration and activation of macrophages, neutrophils and lymphocytes, which may further enhance oxidative stress and tissue injury [40]. Previous studies demonstrated that administration of levosimendan exerts a beneficial effect on immune response and redox-homeostatsis [2], [41]. We demonstrated that levosimendan pretreatment decreased the level of free radicals and improve the antioxidant status of the liver in the “late” and “early” groups, too. In addition, histopathological analysis showed less severe inflammatory cell infiltration in the levosimendan pretreated groups.

Our results suggest that levosimendan pretreatment is associated with an attenuation hepatocyte damage during and after warm ischemia. This phenomenon may be a result of pharmacological preconditioning induced by levosimendan.

Rapidly increased expressions of HSPs are induced by various cellular injuries – such as IR – which play an important role in protective mechanisms of IP [42]. HSPs are intra-cellular chaperones protecting the function as well as the structure of injured proteins. Kume et al. showed that the induction of HSP72 in the liver contributes to the reduction of IR injury irrespective of the type of preconditioning [43]. Hence, we decided to determine the HSP72 expression in the liver. We could not detect, however, significant differences between the levosimendan pretreated and the IR groups. Therefore, HSP72 is unlikely to play an important part in hepatoprotective pharmacological preconditioning induced by levosimendan.

Literature data demonstrate that the activation of the reperfusion injury salvage kinase (RISK) pathway – a common target for IP - plays an important role in the anti-ischemic and anti-apoptotic effect of levosimendan [37]. In addition, levosimendan induces nitric oxide (NO) production [44] and is able to open K_ATP_ channels directly without the activation of the conventional preconditioning signaling pathway [45]. The possible roles of NO and K_ATP_ channels are confirmed by examination with 5-HD (a specific mito-K_ATP_ channel blocker) and N^ω^-nitro-l-arginine methyl ester (l-NAME, a nonspecific NO synthase inhibitor), which ceased the beneficial effect of levosimendan [46]. The above mentioned IP-like effects of levosimendan are related to the stabilization of the mitochondria. Maintenance of mitochondrial integrity in hepatocytes is supported by our study, as well. The NADH-tetrazolium enzymehistochemical analysis showed significantly better mitochondrial function and a minor damage only of the energy-balance when we compared “late” pretreatment animals to the corresponding IR group.

The hepatoprotective effect of levosimendan may also be the consequence of the hemodynamic effect of the drug. Our results, however, failed to support this hypothesis due to insufficient data on hemodynamics We could demonstrate that the applied dose of levosimendan induced a typical cardiac effect only: an increase in heart rate and a decrease in blood pressure, similarly to relevant literature data [47]. Interestingly, reperfusion-induced hypotension was relatively lower and the restoration of the hemodynamic parameters was more effective after levosimendan pretreatment shows that it may be worth conducting further investigations along these lines.

Levosimendan pretreatment was applied in two therapeutic time points in order to mimic the time course of protection following ischemic preconditioning. We found that hepatocellular injury was more severe in the “late” experimental groups. This was supported by histological, immunohistochemical analyses as well as measurements of AST, ALT levels and tissue viability of the liver. Major perioperative stress may explain this phenomenon as a consequence of the two-stage operation. Nevertheless, the “late” levosimendan pretreatment resulted in a more significant improvement in terms of hepatocellular injury as compared with the “early” treatment. This may be explained by the fact that the maximal hemodynamic response after administration of levosimendan can be expected at the end of the first or second day [43].

## Conclusions

We examined the effect of levosimendan pretreatment in a liver IR model *in vivo*. Our results suggest that levosimendan can be potentially effective in the prevention hepatic IR injury. Further experiments should also confirm the beneficial effect of levosimendan prior to consolidation of possible application before extensive liver resection or transplantation in the future.
